# Reversible paraparesis due to extramedullary hematopoiesis

**DOI:** 10.1002/ccr3.7621

**Published:** 2023-06-23

**Authors:** Eti Muharremi, Pavllo Djamandi, Arben Rroji, Jera Kruja

**Affiliations:** ^1^ Faculty of Medicine University of Medicine of Tirana Tirana Albania; ^2^ Neurology Service University Hospital Centre “Mother Theresa” Tirana Albania; ^3^ Neuroimaging Service University Hospital Centre “Mother Theresa” Tirana Albania

**Keywords:** extramedullary hematopoiesis, paraparesis, thalassemia

## Abstract

Extramedullary hematopoiesis should be considered in the differential diagnosis of spastic paraplegia in a patient with thalassemia. Radiotherapy remains the treatment of choice, and regain of motor power is expected.

## Graphical abstract

Sagittal T2 weighted MRI: (A) thoracic paravertebral lesions—active lesions (red arrow) show intermediate signal intensity while older inactive lesions (white arrow) show high signal intensity due to fatty infiltration; (B and C) narrowing of the spinal canal and compression of the medulla along the thoracic spine by lesions inside the spinal canal (small blue arrows); (C) lumbo‐sacral active sites of extramedullary hematopoiesis (small yellow arrows).

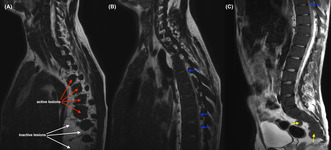

A 34‐year‐old man with thalassemia intermedia presented to our clinic after 3 months of progressive difficulty in controlling the lower limbs. He referred to a similar but milder presentation 1 year ago, which gradually resolved after RBC transfusions and treatment with hydroxyurea.

The patient was icteric on presentation. Spastic paraparesis was present (motor power 2/5), and walking was only possible with bilateral assistance. Patellar reflexes were brisk. Ankle clonus and Babinski response were noted bilaterally. Sensations were decreased below T10 level.

Both CT and MRI of the spine (Figures 1 and 2) revealed paravertebral soft tissue lesions along the whole spine and thoracic lesions compressing the spinal cord, suggestive of extramedullary hematopoiesis. Biopsy was not performed due to the risk of catastrophic hemorrhage.^2^ As per the protocol,^3^ the patient was started on dexamethasone 16 mg/daily for 10 days and he underwent 10 sessions (200 cGy each) of radiotherapy, and four units of RBC were transfused during his hospital stay. Minimal motor deficit was present on discharge, and the patient could walk independently.

**FIGURE 1:**
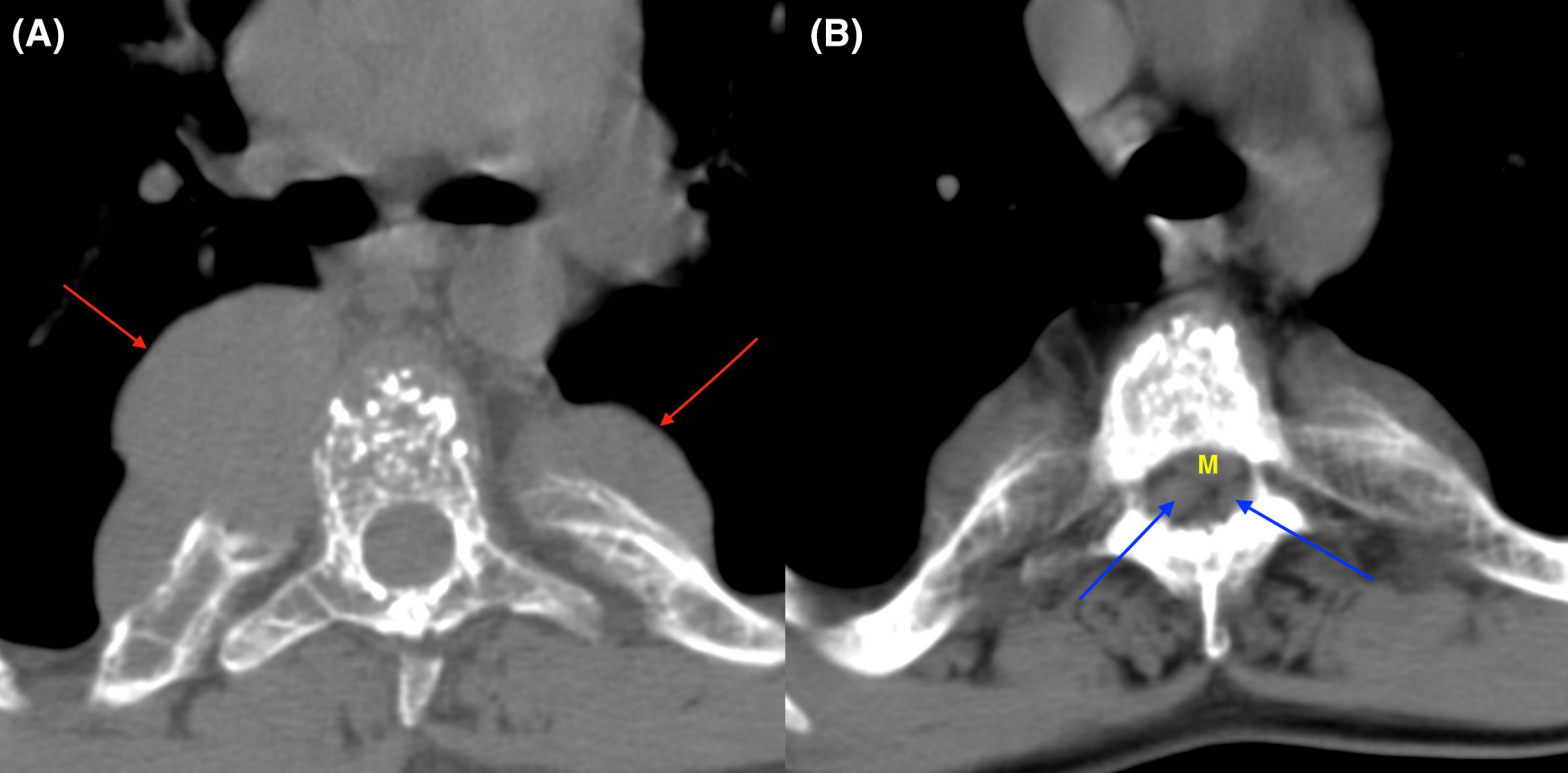
Spine CT: (A) thoracic paravertebral lesions labeled with red arrows; (B) lesions inside the spinal canal at the thoracic level, labeled with blue arrows, narrowing the spinal canal and compressing the medulla (M).

**FIGURE 2:**
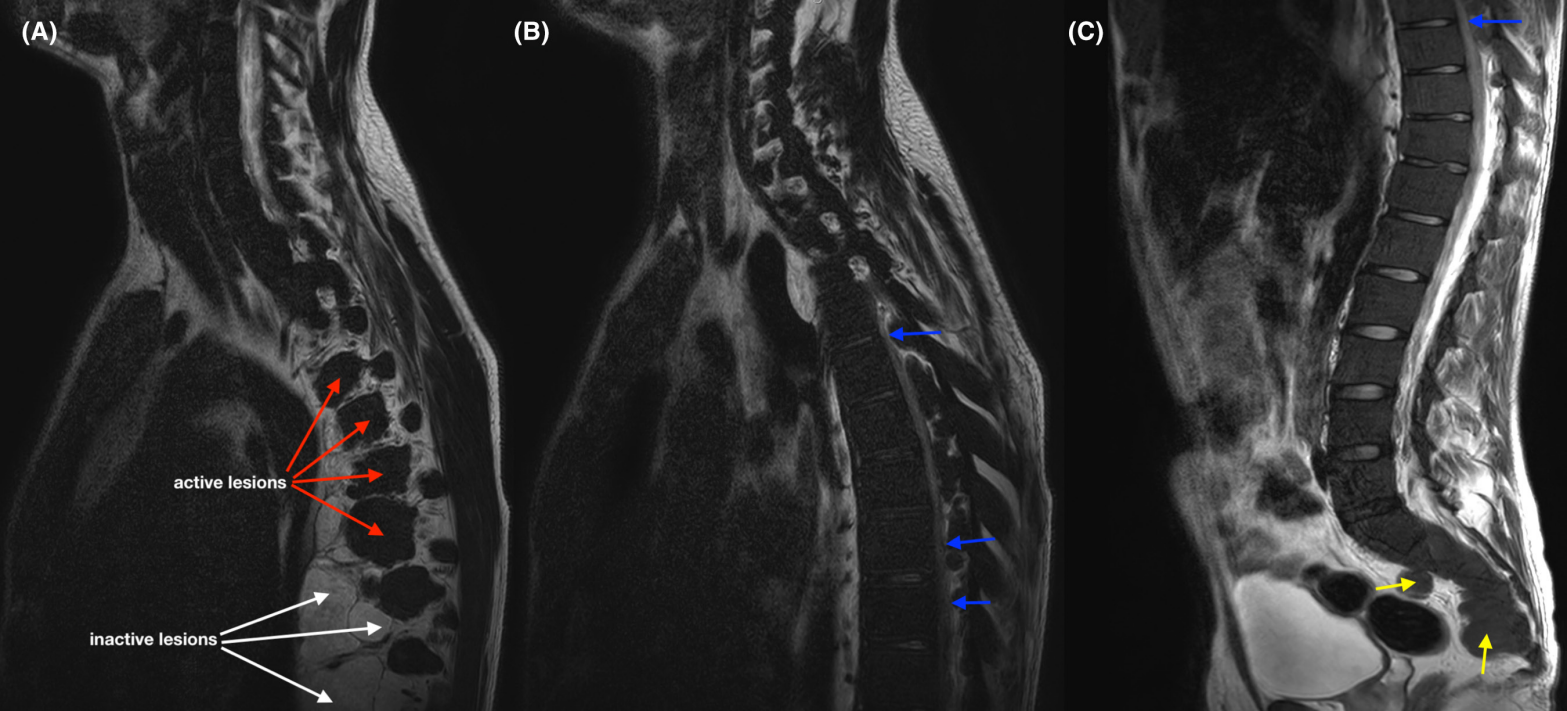
Sagittal T2‐weighted MRI: (A) thoracic paravertebral lesions—active lesions (red arrow) show intermediate signal intensity while older inactive lesions (white arrow) show high signal intensity due to fatty infiltration^1^; (B and C) narrowing of the spinal canal and compression of the medulla along the thoracic spine by lesions inside the spinal canal (small blue arrows); (C) lumbosacral active sites of extramedullary hematopoiesis (small yellow arrows).

## CONSENT STATEMENT

Written informed consent was obtained from the patient to publish this report in accordance with the journal's patient consent policy.

## AUTHOR CONTRIBUTIONS

**Eti Muharremi:** Conceptualization; investigation; project administration; visualization; writing – original draft; writing – review and editing. **Pavllo Djamandi:** Investigation. **Arben Rroji:** Investigation. **Jera Kruja:** Supervision.

## CONFLICT OF INTEREST STATEMENT

None.

## Data Availability

Data sharing not applicable to this article as no datasets were generated or analysed during the current study.

## References

[1] Mesurolle B , Sayag E , Meingan P , Lasser P , Duvillard P , Vanel D . Retroperitoneal extramedullary hematopoiesis: sonographic, CT and MR imaging appearance. AJR Am J Roentgenol. 1996;167:1139‐1140. 8911166 10.2214/ajr.167.5.8911166

[2] Oustwani MB , Kurtides ES , Christ M , Ciric I . Spinal cord compression with paraplegia in myelofibrosis. Arch Neurol. 1980;37:389‐390. 7387474 10.1001/archneur.1980.00500550091019

[3] Haidar R , Mhaidli H , Taher AT . Paraspinal extramedullary hematopoiesis in patients with thalassemia intermedia. Eur Spine J. 2010;19(6):871‐878. doi:10.1007/s00586-010-1357-2 20204423 PMC2899982

